# Tele-Mental Health Service: Unveiling the Disparity and Impact on Healthcare Access and Expenditures during the COVID-19 Pandemic in Mississippi

**DOI:** 10.3390/ijerph21070819

**Published:** 2024-06-22

**Authors:** Yunxi Zhang, Lincy S. Lal, Yueh-Yun Lin, J. Michael Swint, Ying Zhang, Richard L. Summers, Barbara F. Jones, Saurabh Chandra, Mark E. Ladner

**Affiliations:** 1 Department of Data Science, University of Mississippi Medical Center, Jackson, MS 39216, USA; 2 Center for Telehealth, University of Mississippi Medical Center, Jackson, MS 39216, USA; ylin@umc.edu (Y.-Y.L.); schandra@umc.edu (S.C.); 3 Department of Management, Policy and Community Health, The University of Texas School of Public Health, Houston, TX 77030, USA; lincy.lal@uth.tmc.edu (L.S.L.); john.m.swint@uth.tmc.edu (J.M.S.); 4 John P and Katherine G McGovern Medical School, Institute for Clinical Research and Learning Healthcare, The University of Texas Health Science Center at Houston, Houston, TX 77030, USA; 5 Center for Informatics and Analytics, University of Mississippi Medical Center, Jackson, MS 39216, USA; yzhang1@umc.edu; 6 Department of Emergency Medicine, University of Mississippi Medical Center, Jackson, MS 39216, USA; rsummers@umc.edu; 7 Department of Psychiatry and Human Behavior, University of Mississippi Medical Center, Jackson, MS 39216, USA; bfjones@umc.edu (B.F.J.); mladner3@umc.edu (M.E.L.); 8 Department of Medicine, University of Mississippi Medical Center, Jackson, MS 39216, USA

**Keywords:** telehealth, mental health services, access to health care, healthcare resources, health expenditure, healthcare disparities, socioeconomic disparities, health equity

## Abstract

During the COVID-19 pandemic, tele-mental health (TMH) was a viable approach for providing accessible mental and behavioral health (MBH) services. This study examines the sociodemographic disparities in TMH utilization and its effects on healthcare resource utilization (HCRU) and medical expenditures in Mississippi. Utilizing a cohort of 6787 insured adult patients at the University of Mississippi Medical Center and its affiliated sites between January 2020 and June 2023, including 3065 who accessed TMH services, we observed sociodemographic disparities between TMH and non-TMH cohorts. The TMH cohort was more likely to be younger, female, White/Caucasian, using payment methods other than Medicare, Medicaid, or commercial insurers, residing in rural areas, and with higher household income compared to the non-TMH cohort. Adjusting for sociodemographic factors, TMH utilization was associated with a 190% increase in MBH-related outpatient visits, a 17% increase in MBH-related medical expenditures, and a 12% decrease in all-cause medical expenditures (all *p* < 0.001). Among rural residents, TMH utilization was associated with a 205% increase in MBH-related outpatient visits and a 19% decrease in all-cause medical expenditures (both *p* < 0.001). This study underscores the importance of addressing sociodemographic disparities in TMH services to promote equitable healthcare access while reducing overall medical expenditures.

## 1. Introduction

The prolonged duration of the Coronavirus Disease 2019 (COVID-19) pandemic, coupled with the associated stressors, disruptions to daily life, and limited access to healthcare, exacerbated adverse mental health conditions [1,2]. According to the Centers for Disease Control and Prevention (CDC), a considerable number of adults reported symptoms of anxiety or depression, increased substance use, and serious thoughts of suicide during the COVID-19 outbreak [1,2]. Vulnerable populations, including those facing socioeconomic disparities and residing in rural areas, were particularly challenged by this situation [3,4,5,6].

Tele-mental health (TMH) services emerged as a viable solution. TMH leverages telecommunication and videoconferencing technologies to facilitate decentralized mental and behavioral healthcare services, allowing patients in remote locations to overcome the lack of access to healthcare imposed by physical distance. A systematic review examining the role of TMH services, which includes studies from multiple countries such as Austria, Australia, China, the Dominican Republic, Spain, and the United States (U.S), demonstrates that TMH helped reduce the burden of mental health diseases and promoted individual wellbeing during the COVID-19 pandemic [7]. Initially, the Centers for Medicare & Medicaid Services (CMS) telehealth reimbursement policy focused on rural residents, requiring encounters to take place at a clinic or facility in a rural area [8,9,10]. In response to the COVID-19 Public Health Emergency (PHE), CMS expanded its telehealth reimbursement policy to cover a broader range of TMH services, enabling beneficiaries from diverse geographic areas and locations, including their homes, to access TMH services. Other payers, such as United Healthcare and Cigna, also updated their reimbursement policies, including the elimination of cost-sharing for telehealth services [11,12].

Sociodemographic disparities, characterized by factors such as age, race, and socioeconomic status, severely challenge the provision of health equity in mental health care, particularly in underserved populations and rural areas [9,10,13,14,15,16]. Mississippi, a predominantly rural and economically disadvantaged state, faces grave disparities and a shortage of mental health services [17]. With the fourth highest income inequality in the nation, the top 20% of households in Mississippi accounted for 52% of all statewide earnings, while the bottom 20% of households only accounted for 3% of earnings [18,19]. Moreover, Mississippi has the fourth highest rate of uninsured adults with mental illness (18.2%) and a low number of mental health treatment centers (36.33 per 10,000 businesses) [20]. The COVID-19 pandemic and associated lockdown measures further exacerbated mental health conditions in Mississippi, with 44.3% of adults exhibiting symptoms of anxiety or depressive disorder, the highest rate in the nation [21]. Addressing disparities and promoting health equity is crucial in ensuring that all individuals, regardless of sociodemographic characteristics or geographic locations, have access to quality mental health services. 

A body of literature has reported the feasibility and efficacy of TMH in diagnosing and managing mental illness conditions [22,23]. A systematic review further highlighted its wide variety of innovative and inexpensive choices for providers, as well as its value in augmenting primary care and emergency consultations [24]. However, a recent scoping review points to a gap in research concerning disparities in digital equity and the associated healthcare resource utilization (HCRU) and costs [23]. Previous studies among Mississippi Medicare and Medicaid beneficiaries and the general American population have shown the value of TMH in reducing all-cause HCRU and expenditures [12,25,26,27]. A difference-in-difference study of commercially insured American patients further indicates the causal impact of TMH on increasing mental health-related costs without significantly affecting total healthcare costs [28]. However, studies specifically focusing on patients facing sociodemographic disparities and residing in rural areas are still lacking. The objective of this study is to evaluate the usage of TMH services in a rural patient population facing sociodemographic disparities. We aimed to examine the associations between TMH usage and sociodemographic factors, as well as the impact of TMH on all-cause and mental and behavioral health-related HCRU and medical expenditures during the COVID-19 pandemic. By focusing on a specific population within Mississippi, this study provides insights into the potential benefits of TMH services in addressing mental health needs in an underserved, resource-limited setting. 

## 2. Materials and Methods

### 2.1. Ethical Considerations

This study was approved by the University of Mississippi Medical Center (UMMC) institutional review board with a waiver of informed consent. We report this study following the Strengthening the Reporting of Observational Studies in Epidemiology (STROBE) guideline [29].

### 2.2. Study Design, Setting, and Participants

We conducted a retrospective cohort study to compare sociodemographic characteristics, HCRU, and medical expenditures among patients who used TMH services and those who did not at the UMMC between 1 January 2020 and 30 June 2023. 

UMMC, Mississippi’s only academic medical center, provides patient-centered treatment, clinical excellence, and an advanced level of care unavailable anywhere else in the state [30]. It has been at the forefront of mental health care. With the declaration of the COVID-19 PHE, UMMC, including its affiliated sites, transitioned most mental health services to TMH within a week, demonstrating its commitment to maintaining healthcare access during the pandemic. Located in the Jackson metropolitan area, UMMC serves a patient population with various sociodemographic backgrounds, including economically disadvantaged and underserved populations from rural areas.

The study cohort consisted of insured adult patients who regularly sought healthcare from UMMC. This was conducted to minimize potential bias, as patients may seek healthcare services from multiple institutions [31]. Specifically, patients who met the following criteria were included in this study: (1) aged 18 years or older, (2) had at least one mental and behavioral health service primarily paid by insurance, (3) had at least three scheduled visits per year for two years during the study period, and (4) completed at least two visits, either TMH or in-person outpatient, with a gap of at least 3 months between these two visits. Mental and behavioral health-associated encounters were identified through the provider’s academic department, the Department of Psychiatry and Human Behavior at UMMC, along with at least one of the first two diagnosis codes of a visit falling in the F01 to F99 range of the International Classification of Diseases, Tenth Revision (ICD-10). 

Furthermore, the study subjects were categorized into two cohorts based on their utilization of TMH services throughout the study period. Subjects who had completed at least one TMH service were assigned to the TMH cohort, whereas all others were assigned to the non-TMH cohort. We identified TMH services based on the visit type documented for each encounter. Subgroup analysis was conducted to evaluate subjects from rural areas in further detail.

### 2.3. Variables and Data Sources

Medical records were extracted from the UMMC enterprise data warehouse to examine sociodemographic characteristics, HCRU, and medical expenditures between TMH and non-TMH cohorts. Sociodemographic characteristics considered in this study include age, sex, race, primary insurance, rurality, and household income. Age was categorized into four groups: 18 to 34 years, 35 to 49 years, 50 to 64 years, and 65 years or older. Race was categorized into three groups: White/Caucasian, Black/African American, and other. The other race group included subjects identifying as American Indian, Alaska Native, Native Hawaiian, other Pacific Islander, Mississippi Band Choctaw Indian, Asian, Hispanic, Multiracial, and others. Subjects of unknown race or those who refused to provide this information were considered missing. Primary insurance was defined as the insurance most frequently used for mental and behavioral health visits during the study period and was categorized into four groups: commercial insurance, Medicare, Medicaid, and others, including workers’ compensation insurance, managed care, and contractual agreement coverage. Rurality was defined using the Rural–Urban Commuting Area (RUCA) Codes, with codes greater than 3 indicating rural areas. Household income was estimated through the median household income data from the U.S. Census Bureau’s Small Area Income and Poverty Estimates (SAIPE) program [32]. 

HCRU was assessed using mental and behavioral health-related and all-cause outpatient visits, inpatient admissions, and emergency department (ED) visits. Given the variability in payment by insurance and self-pay, the Medicare Physician Fee Schedule (MPFS) was used to estimate the standardized pricing for medical expenditures in Mississippi. Specifically, the facility fee schedule amount for 2023 was applied through Current Procedural Terminology (CPT) and Level II Healthcare Common Procedure Coding System (HCPCS) codes, with locality 00 and carrier 0730200 for Mississippi. Due to the variation in the length of follow-up, HCRU and medical expenditures were reported as per-patient-per-month (PPPM).

### 2.4. Statistical Analysis

Descriptive statistics, including mean with standard deviation (SD) and frequency with percentage (%), were used to summarize continuous and categorical variables. The Shapiro–Wilk test was used to examine the normality of continuous variables for HCRU and medical expenditures. We examined the association between sociodemographic factors and the utilization of TMH services using Pearson’s χ^2^ test. The odds ratio (OR) with its 95% confidence interval (CI) was also reported to present the strength of the association.

The Wilcoxon rank sum test was employed to compare the non-normally distributed variables of HCRU and medical expenditures. To adjust for the sociodemographic factors, generalized linear regression models (GLMs) with log links were constructed to assess the impact of TMH usage on HCRU and medical expenditures. Specifically, we fitted Poisson regression models, negative binomial regression models, or zero-inflated Poisson regression models for HCRU outcomes, depending on their distributions, and Gamma regression models for medical expenditures.

In addition, a subgroup analysis was conducted for subjects residing in rural areas. Sociodemographic characteristics, HCRU, and medical expenditures between TMH and non-TMH cohorts within this subgroup were compared using the same subgroup analysis. In GLMs, we control for all sociodemographic factors except for rurality, as all subjects were from rural areas. 

Statistical significance was determined using two-sided tests with an alpha level of 0.05. All statistical analyses were conducted using SAS statistical software (version 9.4, SAS Institute Inc., Cary, NC, USA).

## 3. Results

### 3.1. Sociodemographic Characteristics

A total of 6787 subjects were included in this study, with 3065 utilizing TMH services and 3722 not. Table 1 presents the sociodemographic characteristics of all subjects and by cohort. The majority of subjects were in the age group of 50 to 64 years (31.87%), female (67.91%), and identified as Black/African American (55.70%). Additionally, the majority had commercial insurance as the primary insurance (40.77%), resided in urban areas (77.97%), and had an annual household income between $42,000 and $50,000 (51.26%). All sociodemographic factors significantly varied between the TMH and non-TMH cohorts, including age, sex, race, primary insurance, rural residency, and household income (all *p* < 0.001).

In the TMH cohort, the largest age group was 35 to 49 years (29.82%), followed by 50 to 64 years (28.74%) and 18 to 34 years (27.86%). In contrast, the largest age group in the non-TMH cohort was 50 to 64 years (34.44%), followed by 35 to 49 years (27.22%) and 18 to 34 (18.57%). While the over-65 age group constitutes the smallest proportion in both cohorts, it was less prominent in the TMH cohort (13.57% vs. 19.77%). Compared to the non-TMH cohort, the TMH cohort had a higher proportion of females (72.76% vs. 63.92%), a higher proportion of White/Caucasian subjects (51.18% vs. 35.48%), a lower proportion of Black/African American subjects (47.17% vs. 62.69%), and a lower proportion of subjects with other races (1.65% vs. 1.84%). Furthermore, the TMH cohort had higher odds of using other insurance than Medicare with an OR of 1.93 (95% CI: 1.60–2.34) and higher odds of residing in rural areas with an OR of 1.22 (95% CI: 1.09–1.37). In terms of household income, the TMH cohort had a similar proportion of subjects with household incomes of less than $42,000 (13.12% vs. 12.60%), a greater proportion with incomes over $50,000 (38.30% vs. 33.93%), but a lower proportion with incomes in the range of $42,000 to $50,000 (48.58% vs. 53.47%), compared to the non-TMH cohort.

### 3.2. HCRU and Expenditures

**Figure 1:**
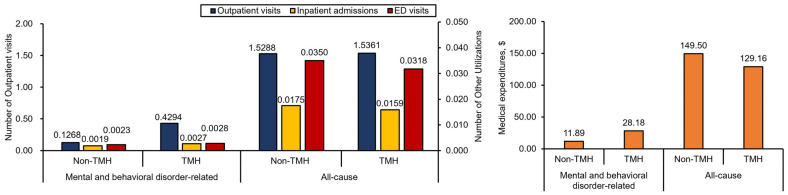
Average mental and behavioral health-related and all-cause HCRU and medical expenditure PPPM.

Compared to the non-TMH cohort, the TMH cohort had significantly more mental and behavioral health-related outpatient visits (mean (SD): 0.43 (0.46) vs. 0.13 (0.31) PPPM), inpatient admissions (mean (SD): 0.0027 (0.02) vs. 0.0019 (0.02) PPPM), ED visits (mean (SD): 0.0028 (0.01) vs. 0.0023 (0.02) PPPM), and medical expenditures (mean (SD): $28.18 (33.26) vs. $11.89 (34.91) PPPM), all with *p* < 0.001. Regarding the all-cause HCRU and medical expenditures, the TMH cohort had lower medical expenditures (mean (SD): $129.16 (176.86) vs. $149.50 (230.43) PPPM; *p* < 0.001) (Figure 1 and Supplementary Table S1). 

After adjusting for sociodemographic factors, TMH utilization was estimated to be associated with a 190% increase in mental and behavioral health-related outpatient visits, a 17% increase in mental and behavioral health-related medical expenditures, and a 12% decrease in all-cause medical expenditures (all *p* < 0.001) (Table 2).

### 3.3. Subgroup Analysis: Subjects Residing in Rural Areas

When considering subjects residing in rural areas, TMH utilization was significantly associated with age (*p* < 0.001), sex (*p* < 0.01), race (*p* < 0.001), and primary insurance (*p* = 0.01). Compared to the non-TMH cohort, the TMH cohort had lower odds of falling into the age groups 50–64 and over 65 when contrasted with the age group 18–34, with odds ratios of 0.54 (95% CI: 0.41–0.70) and 0.52 (95% CI: 0.36–0.74), respectively. Moreover, the TMH cohort had a higher proportion of females (71.35% vs. 61.81%), a higher proportion of White/Caucasian subjects (49.59% vs. 36.40%), and a lower proportion of Black/African American subjects (48.21% vs. 61.89%). In addition, the TMH cohort exhibited higher odds of using other insurance than Medicare, with ORs of 1.97 (95% CI: 1.25–3.09). Furthermore, the TMH cohort had higher proportions of subjects with household incomes of $42,000–$50,000 (37.79% vs. 35.70%) and $50,000 (12.82% vs. 10.76%), but a lower proportion with incomes less than $42,000 (49.39% vs. 53.54%) (Table 3).

**Figure 2:**
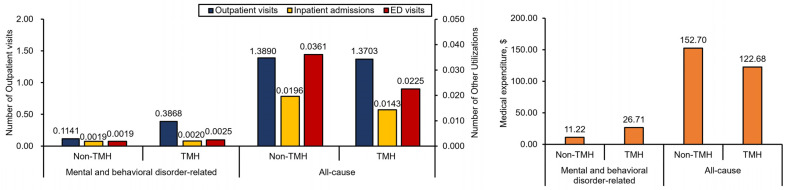
Average mental and behavioral health-related and all-cause HCRU and medical expenditure PPPM of subjects residing in rural areas.

Regarding the unadjusted HCRU and medical expenditures, the TMH cohort residing in rural areas had significantly more mental and behavioral health-related outpatient visits (mean (SD): 0.39 (0.39) vs. 0.11 (0.28) PPPM; *p* < 0.001), inpatient admissions (mean (SD): 0.0020 (0.01) vs. 0.0019 (0.02) PPPM; *p* = 0.01), and medical expenditures (mean (SD): $26.71 (30.30) vs. $11.22 (37.31) PPPM; *p* < 0.001), but lower all-cause medical expenditures (mean (SD): $122.68 (167.04) vs. $152.70 (227.91) PPPM; *p* = 0.002) (Figure 2 and Supplementary Table S2).

After adjusting for all sociodemographic factors, TMH utilization among subjects residing in rural areas was estimated to be associated with a 205% increase in mental and behavioral health-related outpatient visits but a 19% decrease in all-cause medical expenditures (all *p* < 0.001) (Table 4).

## 4. Discussion

### 4.1. Principal Results

Our findings from this study shed light on the sociodemographic characteristics, HCRU, and medical expenditures associated with the utilization of TMH services at a medical center in Mississippi during the COVID-19 pandemic. 

Significant sociodemographic disparities were identified between the TMH and non-TMH cohorts. The TMH cohort had a higher proportion of younger subjects and females, suggesting the appeal and accessibility of TMH services to these groups, which aligns with the increasing acceptance and utilization of telehealth among these populations [33,34]. Moreover, a higher proportion of White/Caucasian subjects in the TMH cohort indicates the potential accessibility of TMH services within this racial group, consistent with studies indicating lower technology usage for health management among older racial minorities [35,36]. Furthermore, the higher proportion of subjects residing in rural areas in the TMH cohort demonstrates the crucial role of TMH services in addressing mental health needs among rural populations and its potential to overcome geographical barriers and improve mental healthcare access in underserved rural communities [8,37]. The primary insurance disparities between TMH and non-TMH cohorts may reflect telehealth business models and insurance coverage policies. Additionally, the TMH cohort included a higher proportion of subjects with household incomes greater than $50,000, implying better access to TMH services in this group, potentially due to factors such as technology availability, insurance coverage, or financial resources. These findings highlight the importance of addressing sociodemographic disparities to achieve equitable access to TMH services, particularly among underserved populations, while considering digital health equity [34,35,38,39].

In terms of HCRU and medical expenditures, the TMH cohort exhibited significantly higher mental and behavioral health-related outpatient visits, inpatient admissions, ED visits, and medical expenditures compared to the non-TMH cohort while experiencing decreased all-cause medical expenditures. After adjusting for sociodemographic factors, TMH utilization remained significantly associated with increased mental and behavioral health-related outpatient visits and medical expenditures. These findings suggest the vital role of TMH services in enhancing access to mental healthcare. Interestingly, TMH utilization was also associated with a decrease in all-cause medical expenditures. The improved access to mental healthcare through TMH services could potentially enhance mental and behavioral health and lifestyles, leading to better health status and consequently reducing overall medical expenditures [40]. These results are consistent with previous studies showing cost savings associated with outpatient behavioral health treatment among populations covered by commercial insurance and those diagnosed with cancers [31,41,42]. The observed reduction in all-cause medical expenditures underscores the potential economic benefits of TMH services, emphasizing the importance of addressing mental health needs to achieve better health outcomes and cost-efficiency. Future research may explore opportunities for integrating TMH and primary care services [43]. 

Furthermore, our subgroup analysis focusing on subjects residing in rural areas revealed significant associations between TMH utilization and sociodemographic factors, particularly age, sex, race, and primary insurance. This highlights the need for targeted efforts to improve access to TMH services among seniors and underserved racial groups, as well as Medicare and commercially insured populations, promoting equitable utilization of TMH resources [35,44]. Moreover, the TMH cohort in rural areas demonstrated higher mental and behavioral health-related outpatient visits and medical expenditures but less mental and behavioral health-related inpatient admissions and all-cause medical expenditures. After adjusting for sociodemographic factors, the significant associations between TMH utilization and increased mental and behavioral health-related outpatient visits, as well as decreased all-cause medical expenditures, persisted. These findings further highlight the value of TMH in improving access to mental healthcare and reducing overall healthcare expenditures in rural communities. Efforts should be made to enhance access and utilization of TMH services among underserved rural populations while exploring strategies to improve the delivery and integration of TMH in rural healthcare systems [34,43,45].

### 4.2. Limitations

Several limitations should be considered when interpreting the findings of this study. First, though we adjusted for sociodemographic factors in this retrospective cohort study, unmeasured factors such as patient comorbidities may influence HCRU and medical expenditure outcomes. Consequently, we cannot definitively address the causal effects of TMH on these outcomes. Future studies employing causal inference methodologies are recommended. Second, the focus on TMH utilization within a single academic medical center in Mississippi during the COVID-19 pandemic may limit the generalizability to other geographic regions or time periods with different healthcare infrastructures, TMH implementation practices, and sociodemographic contexts. Although patients from all UMMC-affiliated sites were included, future studies should consider multiple academic centers or healthcare entities to validate the robustness of findings across different geographic regions and patient populations. Additionally, there is potential for missing data since patients may seek care at multiple institutions. This limitation was mitigated by limiting the study sample to patients who had evidence of regular care visits at UMMC and had evidence of insurance coverage for at least one visit. However, as the study sample consisted of insured patients who regularly seek healthcare from UMMC, it may not represent the entire population of Mississippi, such as those without any healthcare access, thereby limiting generalizability to uninsured populations. Future research should identify and address barriers faced by uninsured populations to provide a more comprehensive understanding of TMH utilization and its impact on HCRU and medical expenditures. 

## 5. Conclusions

Our study contributes to the growing body of evidence supporting the importance of addressing sociodemographic disparities and promoting equitable access to TMH services. By investigating TMH utilization in Mississippi throughout the COVID-19 pandemic, our study highlights significant sociodemographic disparities between TMH and non-TMH cohorts, with younger patients, females, those residing in rural areas, and individuals with higher household incomes being more likely to utilize TMH services. A higher proportion of younger patients, females, and White/Caucasian patients in the TMH cohort was observed across all study subjects and within the subgroup of rural residents. These findings collectively suggest the need to ensure equitable access to TMH services across sociodemographic groups. This study also demonstrates the positive impact of TMH on mental and behavioral health-related outpatient visits and medical expenditures, suggesting its value in enhancing access to mental healthcare and reducing overall healthcare expenditures. Moreover, the subgroup analysis focusing on rural areas underscores the crucial role of TMH in addressing mental health needs among rural populations and providing accessible mental healthcare to patients in underserved rural communities. 

## Figures and Tables

**Table 1:** Sociodemographic characteristics of all study subjects and using TMH.

	All Subjects (*n* = 6787)	Non-TMH (*n* = 3722)	TMH (*n* = 3065)	Odds Ratio (95% CI)	*p* Value
Age, yr, no. (%)					<0.001
18–34	1545 (22.76)	691 (18.57)	854 (27.86)	Ref	
35–49	1927 (28.39)	1013 (27.22)	914 (29.82)	0.73 (0.64, 0.84)	
50–64	2163 (31.87)	1282 (34.44)	881 (28.74)	0.56 (0.49, 0.63)	
≥65	1152 (16.97)	736 (19.77)	416 (13.57)	0.46 (0.39, 0.54)	
Female, no. (%)	4609 (67.91)	2379 (63.92)	2230 (72.76)	1.51 (1.36, 1.67)	<0.001
Race, no. (%)					<0.001
White/Caucasian	2869 (42.55)	1314 (35.48)	1555 (51.18)	Ref	
Black/African American	3755 (55.70)	2322 (62.69)	1433 (47.17)	0.52 (0.47, 0.58)	
Others	118 (1.75)	68 (1.84)	50 (1.65)	0.62 (0.43, 0.90)	
Primary insurance, no. (%)					<0.001
Medicare	2174 (32.03)	1212 (32.56)	962 (31.39)	Ref	
Medicaid	1306 (19.24)	706 (18.97)	600 (19.58)	1.07 (0.93, 1.23)	
Commercial	2767 (40.77)	1591 (42.75)	1176 (38.37)	0.93 (0.83, 1.04)	
Other	540 (7.96)	213 (5.72)	327 (10.67)	1.93 (1.60, 2.34)	
Rural residency, no. (%)	1495 (22.03)	762 (20.47)	733 (23.92)	1.22 (1.09, 1.37)	<0.001
Household income, $, no. (%)					<0.001
≤42,000	871 (12.83)	469 (12.60)	402 (13.12)	Ref	
42,000–50,000	3479 (51.26)	1990 (53.47)	1489 (48.58)	0.87 (0.75, 1.01)	
>50,000	2437 (35.91)	1263 (33.93)	1174 (38.30)	1.08 (0.93, 1.27)	

**Table 2:** Adjusted TMH effects on mental and behavioral health-related and all-cause HCRU and medical expenditure PPPM.

	Mental and Behavioral Health-Related			All-Cause		
	Estimates (Std Err)	Exponentiated Estimates (95% CI)	*p* Value	Estimates (Std Err)	Exponentiated Estimates (95% CI)	*p* Value
Outpatient visits	1.07 (0.06)	2.90 (2.60, 3.24)	<0.001	0.04 (0.02)	1.04 (1.00, 1.08)	0.08
Inpatient admissions	0.09 (0.53)	1.10 (0.39, 3.11)	0.86	−0.14 (0.20)	0.87 (0.59, 1.27)	0.47
ED visits	0.02 (0.50)	1.02 (0.38, 2.73)	0.97	−0.11 (0.14)	0.89 (0.68, 1.17)	0.42
Medical expenditures, $	0.16 (0.03)	1.17 (1.11, 1.25)	<0.001	−0.13 (0.02)	0.88 (0.84, 0.92)	<0.001

**Table 3:** Sociodemographic characteristics of subjects residing in rural areas.

	All Subjects (*n* = 1495)	Non-TMH (*n* = 762)	TMH (*n* = 733)	Odds Ratio (95% CI)	*p* Value
Age, yr, no. (%)					<0.001
18–34	369 (24.68)	158 (20.73)	211 (28.79)	Ref	
35–49	423 (28.29)	193 (25.33)	230 (31.38)	0.89 (0.67, 1.18)	
50–64	510 (34.11)	297 (38.98)	213 (29.06)	0.54 (0.41, 0.70)	
≥65	193 (12.91)	114 (14.96)	79 (10.78)	0.52 (0.36, 0.74)	
Female, no. (%)	994 (66.49)	471 (61.81)	523 (71.35)	1.54 (1.24, 1.91)	<0.001
Race, no. (%)					<0.001
White/Caucasian	637 (42.84)	277 (36.40)	360 (49.59)	Ref	
Black/African American	821 (55.21)	471 (61.89)	350 (48.21)	0.57 (0.46, 0.71)	
Others	29 (1.95)	13 (1.71)	16 (2.20)	0.95 (0.45, 2.00)	
Primary insurance, no. (%)					0.01
Medicare	503 (33.65)	259 (33.99)	244 (33.29)	Ref	
Medicaid	380 (25.42)	195 (25.59)	185 (25.24)	1.01 (0.77, 1.32)	
Commercial	515 (34.45)	274 (35.96)	241 (32.88)	0.93 (0.73, 1.19)	
Other	97 (6.49)	34 (4.46)	63 (8.59)	1.97 (1.25, 3.09)	
Household income, $, no. (%)					0.22
≤42,000	770 (51.51)	408 (53.54)	362 (49.39)	Ref	
42,000–50,000	549 (36.72)	272 (35.70)	277 (37.79)	1.15 (0.92, 1.43)	
>50,000	176 (11.77)	82 (10.76)	94 (12.82)	1.29 (0.93, 1.79)	

**Table 4:** Adjusted TMH effects on mental and behavioral health-related and all-cause HCRU and medical expenditure PPPM of subjects residing in rural areas.

	Mental and Behavioral Health-Related			All-Cause		
	Estimates (Std Err)	Exponentiated Estimates (95% CI)	*p* Value	Estimates (Std Err)	Exponentiated Estimates (95% CI)	*p* Value
Outpatient visits	1.11 (0.13)	3.05 (2.38, 3.90)	<0.001	0.01 (0.05)	1.01 (0.92, 1.10)	0.83
Inpatient admissions	−0.33 (1.22)	0.72 (0.07, 7.92)	0.79	−0.38 (0.42)	0.68 (0.30, 1.54)	0.36
ED visits	−0.08 (1.16)	0.92 (0.10, 8.97)	0.95	−0.55 (0.32)	0.58 (0.31, 1.09)	0.08
Medical expenditures, $	0.11 (0.07)	1.11 (0.98, 1.27)	0.11	−0.21 (0.05)	0.81 (0.73, 0.90)	<0.001

## Data Availability

Due to the presence of protected health information (PHI) and in accordance with IRB regulations, the dataset cannot be made publicly available.

## Supplementary Materials

The following supporting information can be downloaded at: https://www.mdpi.com/article/10.3390/ijerph21070819/s1, Table S1: Unadjusted mental health-related and all-cause HCRU and medical expenditures PPPM, Mean (SD); Table S2: Unadjusted mental health-related and all-cause HCRU and medical expenditures PPPM of subjects residing in rural areas, Mean (SD).
