# Supplementary material for: Tele-Mental Health Service: Unveiling the Disparity and Impact on Healthcare Access and Expenditures during the COVID-19 Pandemic in Mississippi
Source: Int J Environ Res Public Health. 2024 Jun 22;21(7):819. doi: 10.3390/ijerph21070819 (PMC11276461; doi:10.3390/ijerph21070819)
Supplement: Supplementary file 1 [file ijerph-21-00819-s001.zip › ijerph-3060489-supplementary.pdf]

Supplementary Table 1. Unadjusted mental health-related and all-cause HCRU and medical expenditures PPPM, Mean (SD).

	Mental and Behavioral Health-Related			All-cause		
	**Non-TMH (n = 3722)**	**TMH (n = 3065)**	***P* value**	**Non-TMH (n = 3722)**	**TMH (n = 3065)**	***P* value**
Outpatient visits	0.1268 (0.31)	0.4294 (0.46)	<0.001	1.5288 (1.53)	1.5361 (1.44)	0.41
Inpatient admissions	0.0019 (0.02)	0.0027 (0.02)	<0.001	0.0175 (0.05)	0.0159 (0.05)	0.20
ED visits	0.0023 (0.02)	0.0028 (0.01)	<0.001	0.0350 (0.09)	0.0318 (0.10)	0.12
Medical expenditures, $	11.89 (34.91)	28.18 (33.26)	<0.001	149.50 (230.43)	129.16 (176.86)	<0.001

Supplementary Table 2. Unadjusted mental health-related and all-cause HCRU and medical expenditures PPPM of subjects residing in rural areas, Mean (SD).

	Mental and Behavioral Health-Related			All-cause		
	**Non-TMH (n = 762)**	**TMH (n = 733)**	***P* value**	**Non-TMH (n = 762)**	**TMH (n = 733)**	***P* value**
Outpatient visits	0.1141(0.28)	0.3868 (0.39)	<0.001	1.389 (1.29)	1.3703 (1.18)	0.99
Inpatient admissions	0.0019 (0.02)	0.0020 (0.01)	0.01	0.0196 (0.06)	0.0143 (0.04)	0.20
ED visits	0.0019 (0.01)	0.0025 (0.01)	0.06	0.0361 (0.11)	0.0225 (0.07)	0.09
Medical expenditures, $	11.22 (37.31)	26.71 (30.30)	<0.001	152.70 (227.91)	122.68 (167.04)	0.002
